# Steroid Avoidance or Withdrawal Regimens in Paediatric Kidney Transplantation: A Meta-Analysis of Randomised Controlled Trials

**DOI:** 10.1371/journal.pone.0146523

**Published:** 2016-03-18

**Authors:** Huanxi Zhang, Yitao Zheng, Longshan Liu, Qian Fu, Jun Li, Qingshan Huang, Huijiao Liu, Ronghai Deng, Changxi Wang

**Affiliations:** 1 Organ Transplant Center, the First Affiliated Hospital, Sun Yat-sen University, Guangzhou, China; 2 Medical Information Institute, Sun Yat-Sen University, Guangzhou, China; 3 Department of Neurology, the First Affiliated Hospital, Sun Yat-sen University, Guangzhou, China; University of Toledo, UNITED STATES

## Abstract

**Background:**

We combined the outcomes of all randomised controlled trials to investigate the safety and efficacy of steroid avoidance or withdrawal (SAW) regimens in paediatric kidney transplantation compared with steroid-based (SB) regimens.

**Methods:**

A systematic literature search of PubMed, Embase, Cochrane Library, the trials registry and BIOSIS previews was performed. A change in the height standardised Z-score from baseline (ΔHSDS) and acute rejection were the primary endpoints.

**Results:**

Eight reports from 5 randomised controlled trials were included, with a total of 528 patients. Sufficient evidence of a significant increase in the ΔHSDS was observed in the SAW group (mean difference (MD) = 0.38, 95% confidence interval (CI) 0.07–0.68, P = 0.01), particularly within the first year post-withdrawal (MD = 0.22, 95% CI 0.10–0.35, P = 0.0003) and in the prepubertal recipients (MD = 0.60, 95% CI 0.21–0.98, P = 0.002). There was no significant difference in the risk of acute rejection between the groups (relative risk = 1.04, 95% CI 0.80–1.36, P = 0.77).

**Conclusions:**

The SAW regimen is justified in select paediatric renal allograft recipients because it provides significant benefits in post-transplant growth within the first year post-withdrawal with minimal effects on the risk of acute rejection, graft function, and graft and patient survival within 3 years post-withdrawal. These select paediatric recipients should have the following characteristics: prepubertal; Caucasian; with primary disease not related to immunological factors; de novo kidney transplant recipient; with low panel reactive antibody.

## Introduction

Steroids have been widely used in immunosuppressive regimens for paediatric kidney allograft recipients. However, the long-term administration of steroids leads to multiple adverse effects, even at a minimal dose. In paediatric recipients, steroid-induced growth retardation is of particular concern [1].

Attempts have been made to avoid or withdraw steroid therapy in paediatric kidney allograft recipients. Several meta-analyses have evaluated the efficacy and safety of steroid avoidance or withdrawal (SAW) protocols in adult renal transplant recipients [2–7]. The most recent meta-analysis has demonstrated that SAW after renal transplantation increases the risk of acute rejection (AR) but decreases the cardiovascular risk [2]. However, few clinical trials have examined SAW protocols in paediatric kidney transplantation (KTx), and no relevant meta-analysis has been published. Three reviews published by R. Grenda in 2010 and 2011 reported on all previous studies exploring the feasibility of SAW protocols in children with renal allografts [8–10], suggesting that these regimens are safe and beneficial for post-transplant growth. However, the studies were limited as they were mostly conducted at single centres, enrolled only a small number of paediatric patients, and/or were performed retrospectively [8]. Several randomised controlled trials (RCTs) evaluating SAW regimens in paediatric KTx have since been published [11–18]. However, the results are conflicting, and the sample size used in each study was not sufficient to draw robust conclusions. In this meta-analysis, we combined the outcomes of all RCTs to investigate the safety and efficacy of SAW protocols compared with steroid-based protocols in paediatric kidney allograft recipients.

## Patients and Methods

### Inclusion criteria and literature search

RCTs comparing the beneficial and harmful effects of SAW regimens with those of steroid-based regimens in paediatric renal transplant recipients were included. The definition of a child varied among countries, and all definitions were accepted. A systematic literature search of PubMed, Embase, Cochrane Library and the trials registry was performed. Conference abstracts and proceedings were searched using BIOSIS previews. Searches were conducted using MeSH keywords and free-text aliases for corticosteroids, child, KTx and RCTs in each database (S1 Table). No language or publication date restrictions were imposed. The references of the included studies and relevant reviews were scanned for potentially relevant studies that may have been missed in the literature search. The final date for the literature search of each database was August 4^th^, 2015.

### Outcome measures

The primary efficacy outcome was linear growth post-transplant, as indicated by a change in height standardised Z-score (ΔHSDS) from baseline. The primary safety outcome was AR. The secondary outcomes were patient and graft survival, renal graft function (expressed as the estimated glomerular filtration rate (eGFR)) and adverse events, including delayed graft function (DGF), hypertension, new-onset diabetes after transplant (NODAT), hyperlipidaemia, infection and post-transplant lymphoproliferative disorders (PTLDs). All outcomes were analysed at different time points.

### Data collection and analysis

#### Study selection, data extraction and management

Studies were selected independently by two authors according to the inclusion criteria. The studies are referred to by the year of the latest reportand its first author. Data were extracted from the published reports and recorded on specific data collection forms by one author and were then verified by another author. Disagreements were resolved by discussion. Data from reports originating from the same clinical trials were combined for analysis. When data could not be pooled directly for meta-analysis, they were transformed using the formulas recommended by the Cochrane Handbook for Systematic Reviews of Interventions [19]. The following information was extracted from each included study: (1) patient characteristics (including age, Tanner stage, cause of ESRD, and pre-transplant panel reactive antibody); (2) intervention type (including dose, duration and frequency of corticosteroid use in the SAW and steroid-based regimens and concomitant immunosuppressive therapy); and (3) outcome measure.

#### Assessment of risk of bias

The Cochrane Collaboration’s tool for assessing risk of bias was applied independently by two authors [20]. Information for judging the risk of bias was collected from all reports originating from one study, as well as the protocol published in the registry, if applicable. Any disagreement was resolved by discussion.

#### Statistical methods

Relative risk (RR) was used as a summary statistic for dichotomous data, and the weighted mean difference (WMD) was used for continuous data. The hazard ratio (HR) was used for time-to-event data. The rate ratio was used in instances in which it was possible for participants to experience more than one event. Statistical heterogeneity among studies was quantified using the I^2^ test [21]. Intention-to-treat (ITT) data were given priority during analysis. If ITT data were not provided, any subsets of data reported by the authors were accepted. The effect sizes of all outcomes were summarised using the random effects model (RE) and the DerSimonian and Laird method. If the I^2^ value was less than 50%, then the fixed effect model (FE) was applied. The Mantel–Haenszel method was used to calculate the RR, and the inverse variance method was used to determine the WMD. The generic inverse-variance method was used to calculate the HR and rate ratio [21]. All summary effects are presented with 95% confidence intervals (CIs). Unless otherwise noted, a P≤0.05 served as the threshold for statistical significance. All analyses were performed with Review Manager (RevMan) ([computer program] Version 5.2, Copenhagen: The Nordic Cochrane Centre, the Cochrane Collaboration, 2012). If data pooling was unavailable for meta-analysis, we provided a qualitative description. A funnel plot was created to assess reporting bias [22].

#### Subgroup analyses

Subgroup analyses were performed based on the developmental statuses of the paediatric recipients (as determined by Tanner staging [23] or an equivalent method) and the time of initiation of steroid withdrawal. The results for the different subgroups were compared using the method described by Borenstein et al. [24], which was implemented with RevMan v5.2.

#### Sensitivity analysis

Sensitivity analysis was performed by including or excluding studies without ITT analyses of all outcomes. We also compared the outcomes obtained from the random effect and fixed effect model.

#### Quality of evidence

The quality of evidence was evaluated using the GRADE approach, which specifies four levels of quality [25,26].

#### Trial sequential analysis

Trial sequential analysis (TSA) was performed with TSA Viewer Version 0.9 Beta ((c) Copyright Copenhagen Trial Unit, 2011) to control for the risk of errors for a given outcome and to help clarify whether additional trials are required. The statistical method was described in the following literatures [27–30]. More information is provided in supporting information (S2 File).

#### Zero-event studies

RevMan 5 was not designed to analyse studies with no events in either intervention group in meta-analyses performed to determine relative risks. Exclusion of zero-event studies was unjustified and may have inflated the magnitude of the pooled treatment effects [31]. Therefore, we performed random effect meta-analysis with an empirical continuity correction of 0.01 for zero-event studies using TSA Viewer [27].

## Results

### Description of studies

#### Results of the search

**Fig 1:**
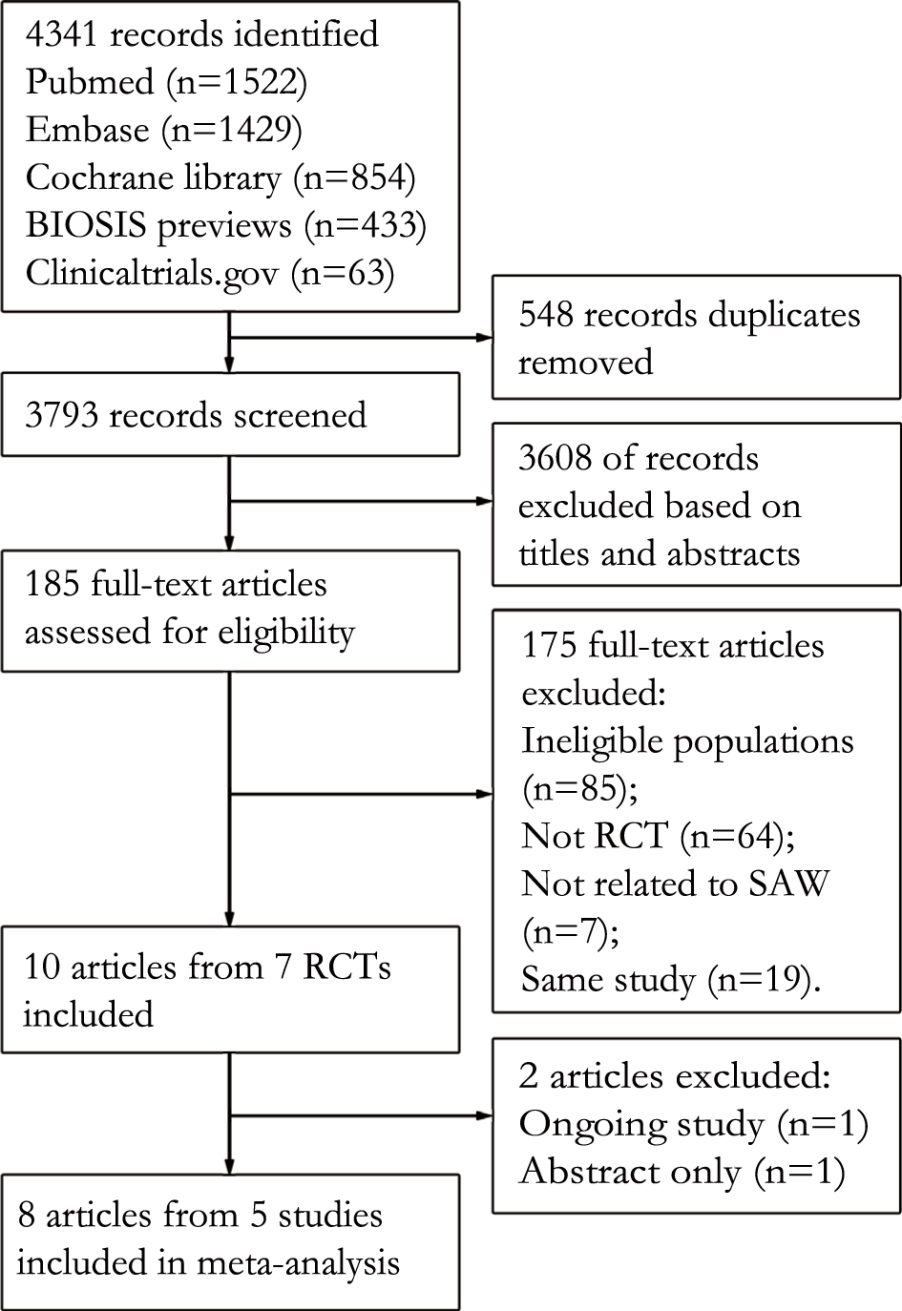
Flow chart showing the sources and identification of the included studies. Abbreviations: RCT, randomised controlled trial; SAW, steroid avoidance or withdrawal.

The article selection process is summarised in Fig 1. Ten reports from 7 randomised clinical trials met the inclusion criteria. However, one on-going RCT [32] and one completed RCT [33] reported only abstracts without sufficient data for analysis and were thus excluded. Finally, 8 reports from 5 RCTs with a total of 528 patients were included [11–18].

#### Included studies

The details of the five included studies are presented in Table 1. All included studies followed a randomised parallel group design. The follow-up periods ranged from 1 to 3 years, and all studies were performed at multiple centres. Three studies used blocked randomisation stratified by pubertal status [11,16,17]. Primary diseases that are not related to immunological factors, such as cystic kidney disease, dysplasia, obstructive uropathy and reflux nephropathy, accounted for 37–92% of the ESRD cases, as determined based on the data from the three studies that reported ESRD causes [11,12,17].

**Table 1:** Characteristics of the included studies.

Source	Follow-up (years post-withdrawal)^c^	Sample size (SAW/SB)	Age range (years)	Country	Race (white/black/Asian/others)(%)	PRA	Time of starting withdrawal post-transplant	Concomitant IST	Primary outcome
Webb et al., 2015[16]	2	98/98	2~18	EU, Israel, South Africa	86.2/5.1/6.9/1.5	≤50%	Day 5	DcL/TAC/MMF	growth
Mericq et al., 2013[11]	1	14/16	1~16	Chile	All Chileans	no limits	Day 6	BsL/TAC/MMF	growth
Sarwal et al., 2012[12]	3	60/70	0~21	USA	52.3/26.1/6.1/15.4	≤20%	CA	DcL/TAC/MMF	growth, AR
Hocker et al., 2010[17]	2.25	23/17	0~18	Germany	97.6/-/2.4/-	≤80%	Year 1–2^a^	CsA/MMF	growth
Benfield et al., 2010[14]	2.5	73/59	0~20	USA	75.0/15.2/3.8/6.1	no limits	Month 6^b^	BsL/TAC/SRL BsL/CsA/SRL	growth

Studies are labelled by the first author and year of the latest fully peer-reviewed publication. PRA, panel-reactive antibody; IST, immunosuppressive therapy; BsL, basiliximab; TAC, tacrolimus; MMF, mycophenolate mofetil; USA, United States of America; EU, European Union; CA, complete avoidance; DcL, daclizumab; AR, acute rejection; UK, United Kingdom; CsA, cyclosporine; SAW, steroid avoidance or withdrawal; SB, steroid-based; SRL, sirolimus.

**a.** Steroid withdrawal began anytime between 1 and 2 years post-transplant and ended 12 weeks later.

**b.** Steroid withdrawal began at 6 months post-transplant and ended by the end of 12 months post-transplant.

**c.** Calculated from the time point of starting steroid withdrawal.

### Risk of bias in the included studies

The mean difference in the ΔHSDS indicated a low risk of bias for all five studies; thus, the summary effect was also at a low risk of bias. The RR of AR was at a low risk of bias for two studies and at an unclear risk for three studies, suggesting plausible bias in the summary effect. Details on the assessment of risk of bias are provided in supporting information (S1 Fig and S1 File). All studies included ITT analyses. The clinical trial conducted by M. R. Benfield et al. was permanently terminated due to an unanticipated high incidence of PTLDs; thus, only data obtained from the randomised patients before trial cessation were included in our study [14].

### Effects of interventions

#### Growth

**Fig 2:**
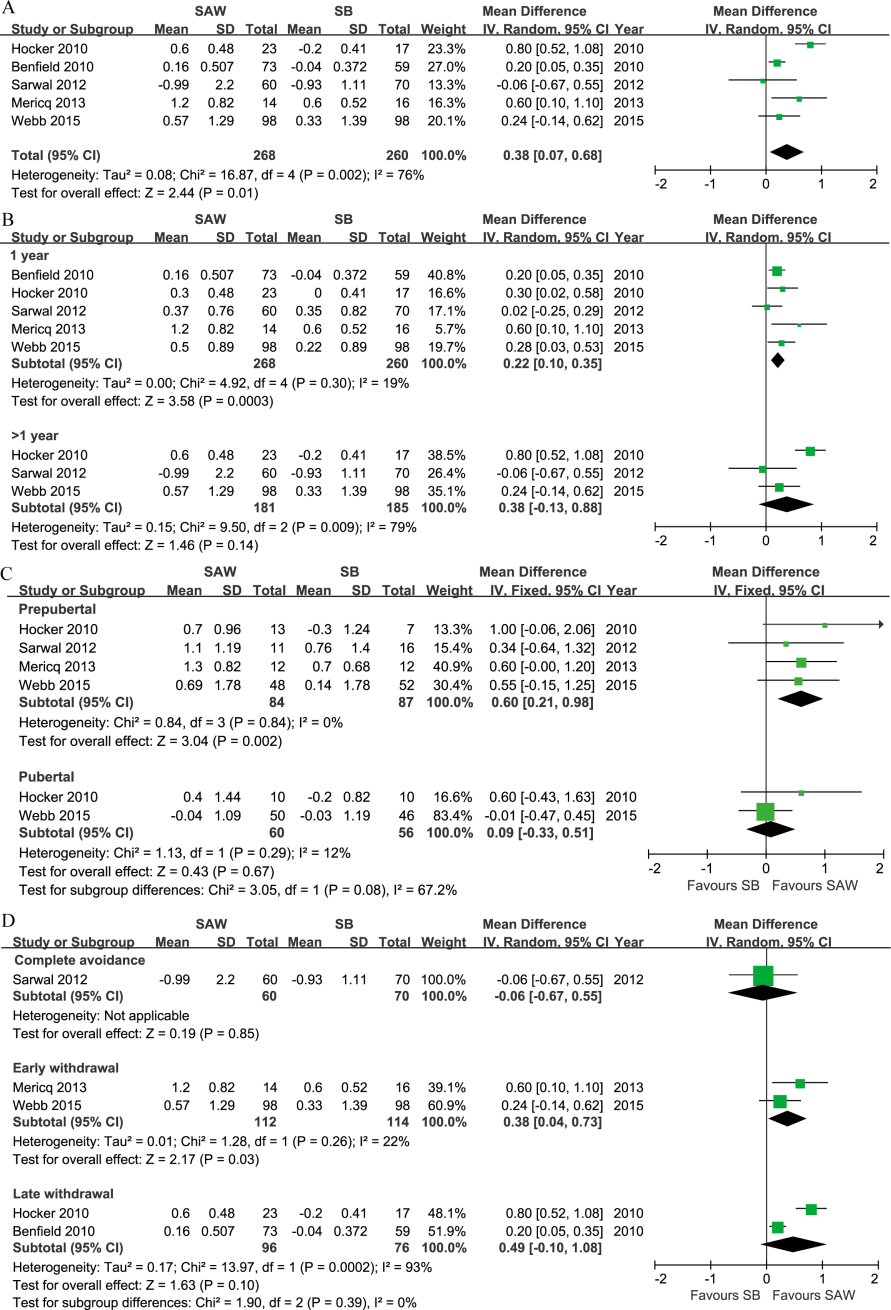
Forest plot comparing the change in height z-score between the SAW and SB groups of paediatric renal allograft recipients. A. No stratification; B. comparison at 1 year and over 1 year post-withdrawal; C. stratified by developmental status; D. stratified by the time of initiation of steroid withdrawal. Abbreviations: ΔHSDS, height z-score change; SAW, steroid avoidance or withdrawal; SB, steroid-based; SD, standard deviation; IV, inverse variance; CI, confidence interval. Produced by RevMan Version 5.2.

All outcomes are summarised in Table 2. RE analysis indicated a significant increase in the ΔHSDS in the SAW group compared with the steroid-based group (WMD = 0.38) (Fig 2A), with a high level of heterogeneity (I^2^ = 76%). The funnel plot was grossly symmetrical (S2 Fig), indicating a lack of reporting bias. An increase in the ΔHSDS was observed in the SAW group at 1 year post-withdrawal (WMD = 0.22) (Fig 2B). However, there was no significant difference after 1 year post-withdrawal (Fig 2B). The funnel plot was grossly symmetrical (S3 Fig).

**Table 2:** Summary of findings.

Outcomes	Effect size [95% CI]	Heterogeneity (I^2^)/%	Number of participants	Quality of evidence (GRADE)	TSA^b^
ΔHSDS	WMD 0.38 [0.07, 0.68]	76	528	High	Efficacy area
ΔHSDS at 1 year post-withdrawal	WMD 0.22 [0.10, 0.35]	19	528	High	Efficacy area
ΔHSDS over 1 year post-withdrawal	WMD 0.38 [-0.13, 0.88]	79	366	Moderate	Not reached
ΔHSDS in prepubertal	WMD 0.60 [0.21, 0.98]	0	171	High	Efficacy area
ΔHSDS in prepubertal at 1 year post-withdrawal	WMD 0.39 [0.16, 0.63]	0	171	High	Efficacy area
ΔHSDS in pubertal	WMD 0.09 [-0.33, 0.51]	12	116	High	Not reached
AR	RR 1.04 [0.80, 1.36]	44	528	High	Futility area
AR at 1 year post-withdrawal	RR 1.12 [0.80, 1.56]	0	528	High	Futility area
AR over 1 year post-withdrawal	RR 1.20 [0.89, 1.60]	30	366	High	Not reached
eGFR at 6 months post-withdrawal	WMD -0.09 [-7.38, 7.20]	0	236	High	Futility area
eGFR at 1 year post-withdrawal	WMD -3.56 [-9.97, 2.86]	6	302	High	Required information size
eGFR at 2 years post-withdrawal	WMD -0.93 [-11.20, 9.34]	0	170	High	Futility area
NODAT	RR 0.31 [0.13, 0.75]	0	326	High	Not reached
Hypertension	RR 0.64 [0.46, 0.88]	0	326	High	Not reached
Infection^a^	RR 1.09 [0.93, 1.28]	0	326	High	Not reached
CMV infection	RR 1.42 [0.88, 2.31]	0	326	Moderate	Not reached
PTLD	RR 1.64 [0.43, 6.25]	47	458	Moderate	Not reached
DGF	RR 1.46 [0.29, 7.41]	55	326	Low	Not reached
Anaemia	RR 1.79 [0.87, 3.69]	63	326	Low	Not reached

a. Comparison of the risk of at least one type of infection.

b. If the cumulative Z curve did not cross the required information size but it crossed the efficacy boundary, then the outcome reached the efficacy area. If the cumulative Z curve did not cross the required information size but it crossed the futility boundary, then the outcome reached the futility area. If the cumulative Z curve crossed neither the required information size nor the efficacy and futility boundary, then the outcome was considered *not reached*.

CI, confidence interval; TSA, trial sequential analysis; HSDS, height standard deviation score; WMD, weighted mean difference; AR, acute rejection; RR, relative risk; eGFR, estimated glomerular filtration rate; NODAT, new-onset diabetes after transplant; PTLDs, post-transplant lymphoproliferative disorders; DGF, delayed graft function.

Following stratification by developmental status, the ΔHSDS was significantly higher in the prepubertal patients undergoing the SAW regimen (WMD = 0.64) (Fig 2C), and the funnel plot was grossly symmetrical (S4 Fig). Further, no significant differences were identified among these patients (Fig 2C). However, a significant difference was observed between the two subgroups (P = 0.05). In addition, there was a significant increase in the ΔHSDS at 1 year post-withdrawal in the prepubertal recipients in the SAW group compared with those in the steroid-based group (RE, WMD = 0.39, 95% CI 0.16–0.63, P = 0.001, I^2^ = 0%).

When the subgroups were stratified by the time of initiation of steroid withdrawal, an increase in the ΔHSDS was identified in the SAW group compared with the steroid-based group in the early withdrawal subgroup (Fig 2D). However, no significant differences in the ΔHSDS were observed in the avoidance or late withdrawal subgroup. A significant test also showed that there were no significant differences in the summary estimate (namely the difference in the ΔHSDS between the SAW and steroid-based groups) among these three subgroups.

#### Acute rejection

**Fig 3:**
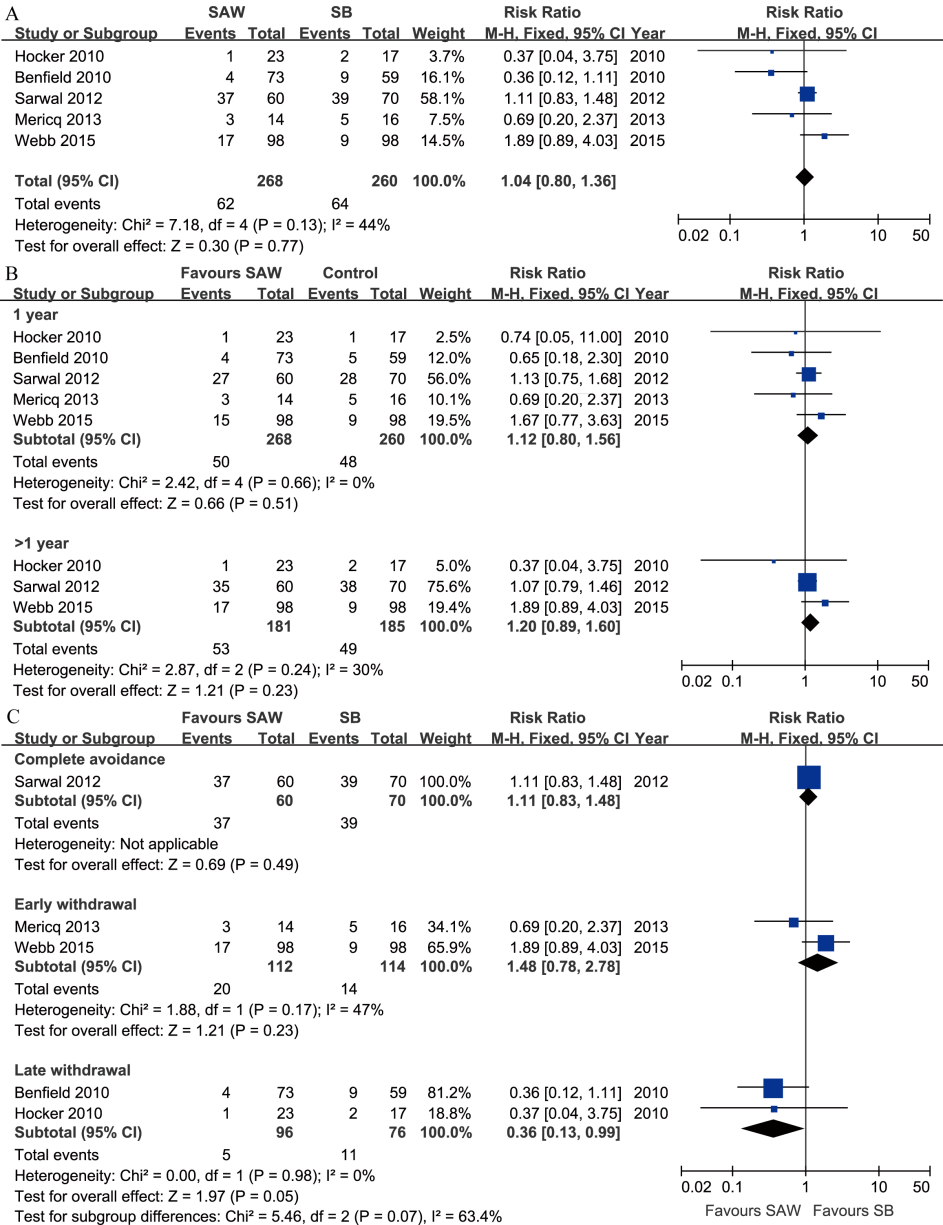
Forest plot comparing the risk of AR between the SAW and SB groups of paediatric renal allograft recipients. A. No stratification; B. comparison at 1 year and over 1 year post-withdrawal; C. stratified by the time of initiation of steroid withdrawal. Abbreviations: AR, acute rejection; SAW, steroid avoidance or withdrawal; SB, steroid-based; M-H, Mantel-Haenszel; CI, confidence interval. Produced by RevMan Version 5.2.

All five studies reported the rate of AR, which was confirmed by renal biopsy (performed if indicated or according to the established protocol). Clinically defined AR was also combined with biopsy-proven AR in the meta-analysis. The rates of T-cell-mediated and antibody-mediated AR, as well as borderline changes of AR, were summed for analysis. Overall, there was no significant difference in the risk of AR between the SAW and steroid-based regimens (Fig 3A). The funnel plot was grossly symmetrical (S5 Fig). At 1 year post-withdrawal, the risk of AR for the SAW regimens was similar to that of the control (Fig 3B).

No adequate AR data stratified by developmental status were provided in any of the five studies; thus, meta-analysis could not be performed. However, subgroup analysis of all studies indicated that there were no significant differences between the SAW and steroid-based groups within either the prepubertal or pubertal subgroup [11, 16,17].

Subgroup analysis based on the time of initiation of steroid withdrawal revealed a significantly reduced risk of AR in the recipients with late steroid withdrawal (Fig 3C).

#### Graft function

**Fig 4:**
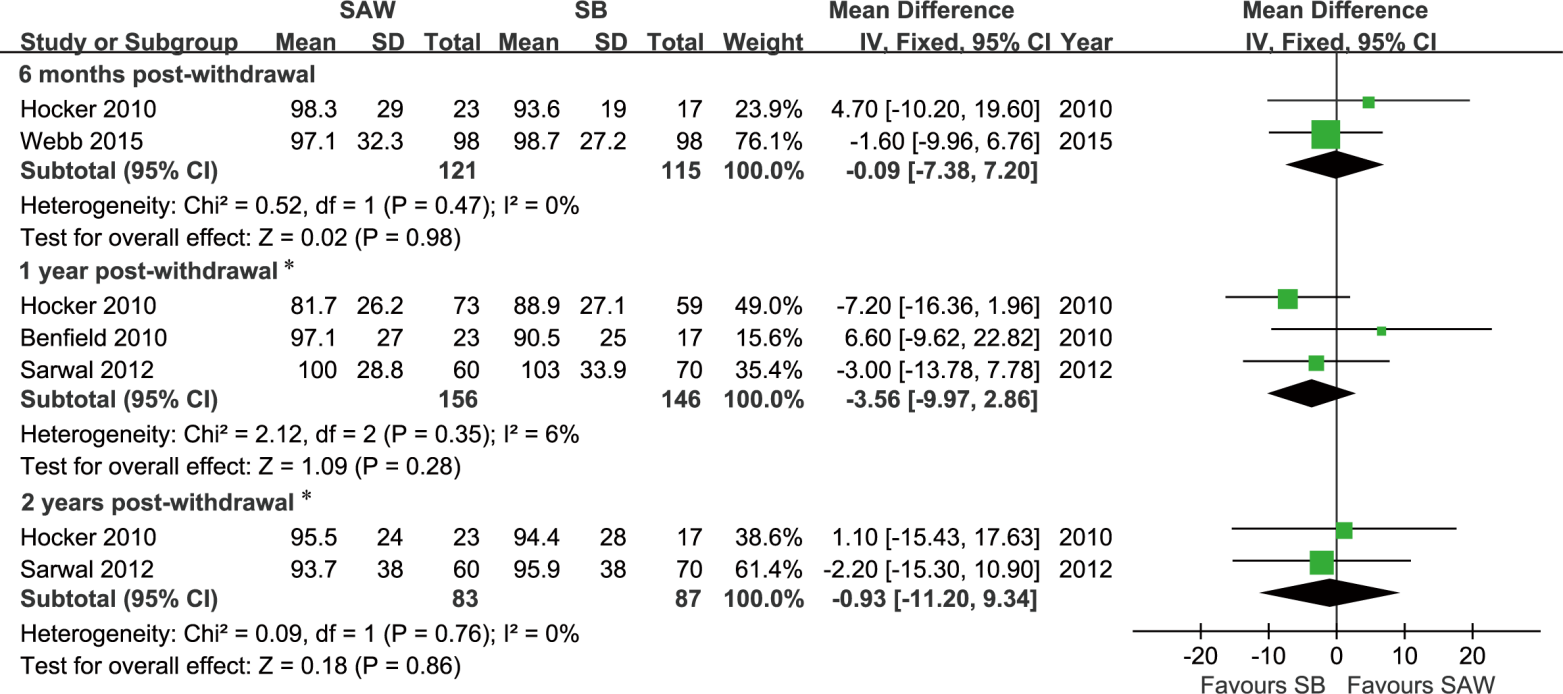
Forest plot comparing the estimated glomerular filtration rate between the SAW and SB groups of paediatric renal allograft recipients at different time points post-withdrawal. A. 6 months; B. 1 year; and C. 2 years. Abbreviations: eGFR, estimated glomerular filtration rate; SAW, steroid avoidance or withdrawal; SB, steroid-based; SD, standard deviation; IV, inverse variance; CI, confidence interval. * Data from the TWIST study (Webb 2015) were excluded because the eGFR distribution was likely skewed at 1 or 2 years after withdrawal; thus, it was inappropriate to convert the interquartile range into a standard deviation. Produced by RevMan Version 5.2.

Renal allograft function, as indicated by the eGFR, which was calculated using the Schwartz formula, was reported as an outcome in 4 studies [11,12,16,17]. There were no significant differences in graft function based on the eGFR between the SAW and steroid-based groups at 6 months post-withdrawal (FE, WMD = -0.09 ml/min/1.73 m^2^, 95% CI -7.38–7.20, P = 0.98, I^2^ = 0%), but significant differences were observed at 1 year post-withdrawal (FE, -4.59 ml/min/1.73 m^2^, 95% CI -8.27–-0.92, P = 0.01, I^2^ = 0%) and 2 years post-withdrawal (FE, -5.57 ml/min/1.73 m^2^, 95% CI -10.55–-0.60, P = 0.03, I^2^ = 0%). The eGFR distribution in the TWIST study was likely skewed at 1 or 2 years after withdrawal [16], and the differences in this rate between the SAW and steroid-based groups became insignificant at 1 year and 2 years post-withdrawal when this study was excluded (Fig 4). All funnel plots were grossly symmetrical.

#### Patient and graft survival

The patient and graft survival data reported by the five studies were not sufficient for estimating HRs due to the lack of event distribution (Table 3). In all studies except those by M. R. Benfield [11,12,16,17], no significant differences in either patient or graft survival between the SAW and steroid-based groups were reported at 1 year (4 studies), 2 years (2 studies) or 3 years (2 studies) post-transplant. In M. R. Benfield’s study [14], a significant increase in the composite of patient and allograft survival at three years post-transplant was reported in the SAW group (98.6%) compared with the steroid-based group (84.5%). Therefore, 1-year, 2-year and 3-year patient and graft survival did not appear to be reduced in the renal recipients receiving the SAW protocol.

**Table 3:** Summary of survival data.

		1 year post-transplant		2 years post-transplant		3 years post-transplant	
Source	Number of participants (SAW/SB)	Graft loss (SAW/SB)	Patient death (SAW/SB)	Graft loss (SAW/SB)	Patient death (SAW/SB)	Graft loss (SAW/SB)	Patient death (SAW/SB)
Webb et al., 2015[16]	98/98	5/3	2/0	5/5	2/0	-/-	-/-
Mericq et al., 2013[11]	14/16	0/0	0/0	-/-	-/-	-/-	-/-
Sarwal et al., 2012[12]	60/70	2/1	0/0	-/-	-/-	3/7	0/0
Hocker et al., 2010^a^[17]	23/17	0/0	0/0	0/0	0/0	-/-	-/-
Benfield et al., 2010[14]	73/59	-/-	-/-	-/-	-/-	1/4	0/5

a. Note that in this study, steroid withdrawal was started at any time between 1 and 2 years post-transplant and completed 12 weeks later. The report provided survival data at the end of 27 months of follow-up. The data are revised here.

SAW, steroid avoidance or withdrawal; SB, steroid-based.

### Adverse events

**Fig 5:**
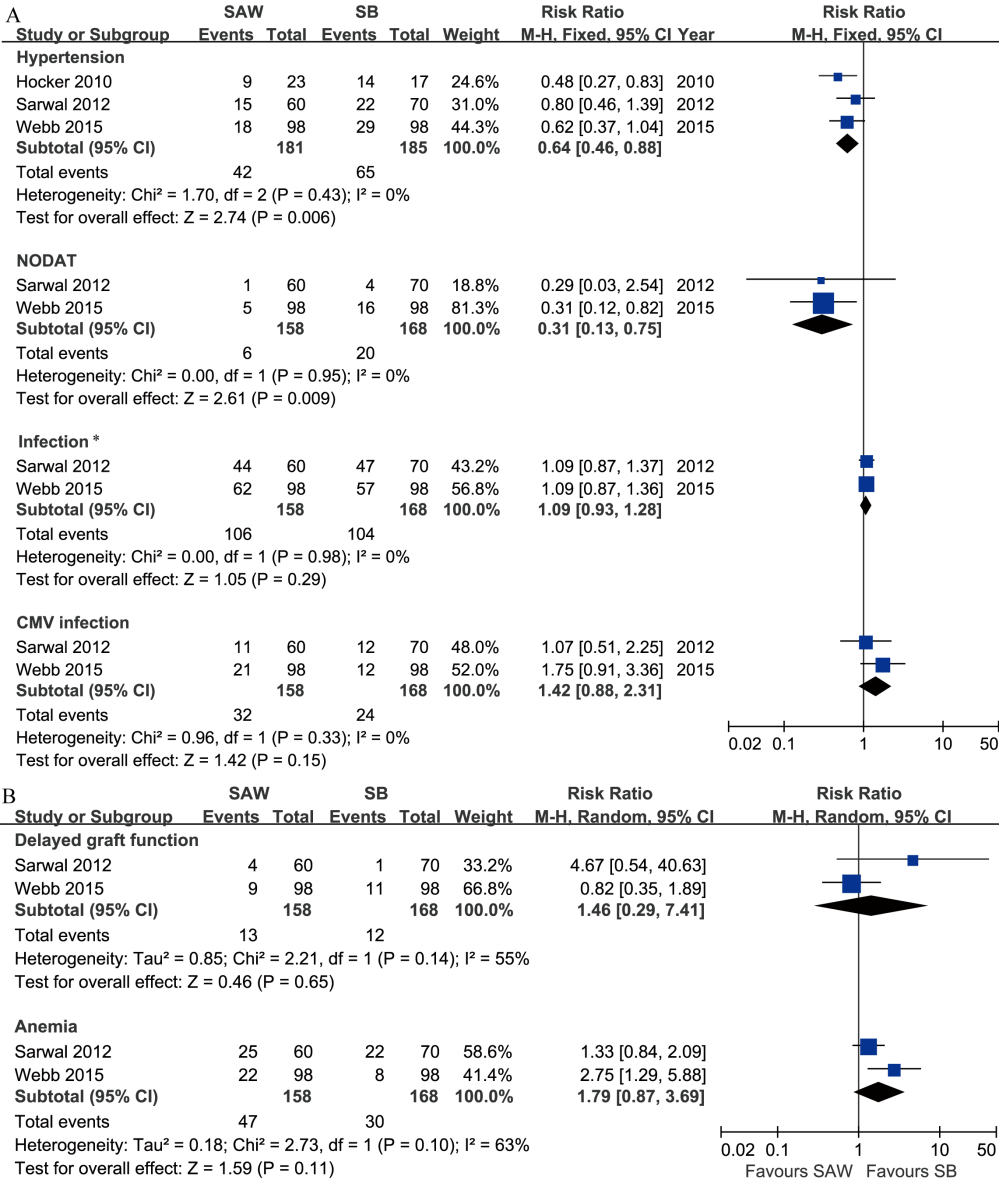
Forest plot comparing the risk of adverse events between the SAW and SB groups of paediatric renal allograft recipients. A. Hypertension, NODAT, infection and CMV infection (fixed effects model, Mantel-Haenszel method); B. delayed graft function and anaemia (random effects model, Mantel-Haenszel method). *Comparison of the risk of at least one type of infection. Abbreviations: NODAT, new-onset diabetes after transplant; CMV, cytomegalovirus; SAW, steroid avoidance or withdrawal; SB, steroid-based; M-H, Mantel-Haenszel; CI, confidence interval. Produced by RevMan Version 5.2.

The follow-up period for assessing adverse events ranged from 1 to 3 years. Significant reductions in the risks of NODAT and hypertension were observed in the SAW group compared with the steroid-based group (Fig 5A). Quantitative data on the serum cholesterol and triglyceride levels were reported in 3 studies but were insufficient for data pooling [11, 16,17]. Data on PTLDs were reported in three studies [12,14,16]. In M. M. Sarwal’s study, no PTLD cases were reported in the SAW or steroid-based group [12]. After continuity correction, no significant differences were identified (3 studies, 458 patients, RE, RR = 1.64, CI 0.43–6.25, P = 0.47, I^2^ = 0%). The results for other adverse events are presented in Fig 5.

### Quality of evidence and trial sequential analysis

The results of the evidence quality assessment and TSA are both summarised in Table 2. TSA revealed that the results obtained by pooling the sample so that it was greater than the *required information size* were trustworthy. The results within either the *efficacy area* or *futility area* were also trustworthy, even though the pooled sample size did not reach the required information size. Further studies are required to confirm the reliability of the other results (labelled as *not reached*). Details on the evidence quality assessment and TSA are provided in supporting information (S6–S9 Figs, S2 Table and S2 File).

## Discussion

Overall, this study has obtained sufficient evidence to conclude that the SAW regimen results in superior catch-up growth post-transplant compared with the steroid-based regimen in paediatric renal allograft recipients. The catch-up growth of the paediatric recipients of all ages was largely due to growth of the prepubertal recipients, and we did not observe superior post-transplant growth of the pubertal recipients receiving the SAW regimen. However, the evidence acquired in the latter comparison was not sufficiently robust. Within the first year post-withdrawal, superior growth was observed for the SAW regimen compared with the steroid-based regimen (sufficient evidence), whereas the effect appeared to be lost over 1 year post-withdrawal (insufficient evidence). In addition, sufficient evidence was obtained to conclude that the SAW regimen did not increase the risk of AR or damage graft function within one to three years after steroid withdrawal. Further, the SAW regimen did not decrease graft or patient survival, and it likely reduced the risks of hypertension and NODAT and therefore the cardiovascular risk. However, the evidence was not sufficiently robust.

The results of this study should be considered with caution in clinical practice due to inconsistencies among the study subjects. More than 70% of the participants were Caucasian, approximately 12% were African, and less than 5% were Asian. Variations in genetic background across races are likely to alter the outcome of the SAW protocol. Moreover, a reduced panel of reactive antibodies (PRA) was required in the recipients prior to KTx in two studies (≤20% [12] or ≤50% [16]), whereas the inclusion criteria were broad (≤80% [17] or had no limitations [11,14]) in the other studies, in which the PRA distribution across participants was not reported. HLA matching was reported in only one study [12]. The majority of recipients received a primary kidney transplant. Moreover, the primary diseases were not related to immunological factors in most recipients in the included studies and thus the risk of recurrence of renal diseases such as glomerulonephritis was reduced. The effect of steroids on prevention of recurrence of renal disease may be underestimated. It was reported that recurrence of glomerulonephritis was the second reason (about 35%) beyond rejection, to reintroduce steroids in steroid-free children post-transplant [34]. It suggested that we should be cautious to apply the SAW regimen to the children with glomerulonephritis as their primary disease. However, this report did not answer directly the question whether the SAW regimen will increase the risk of glomerulonephritis recurrence. Another report showed that steroids withdrawal did not increase the risk of glomerulonephritis recurrence [35]. However, the conclusion was not that robust because it resulted from a respective historically controlled study. In addition, all paediatric recipients (74/74) in the steroids withdrawal group received Mycophenolate mofetil besides cyclosporine while most recipients (56/69) in the steroids-based group received azathioprine, which was likely to bias the result. More robust data are required before stronger conclusions can be drawn.

The magnitude of post-transplant growth in the prepubertal recipients receiving the SAW regimen was greater than that in the pubertal recipients. This increase was attributed in part to the greater potential for growth, shorter duration of ESRD and different causes of ESRD in the prepubertal recipients. Moreover, there was less heterogeneity within the prepubertal and pubertal subgroups, whereas that among all paediatric recipients was large. A similar phenomenon was observed in subgroup analysis based on either different time points post-withdrawal or different times of initiation of steroid withdrawal. These results suggest that the heterogeneity in the post-transplant growth results can be explained partly by differences in developmental status, the time after steroid withdrawal and the time of initiation of steroid withdrawal.

The current meta-analysis demonstrated no increase in the overall risk of AR, in contrast with the significant increase observed in the SAW group in a previous meta-analysis of adult renal transplant recipients (RE, RR = 1.56, 95% CI 1.31–1.87, P<0.0001) [2]. The differences between our study and the previous meta-analysis of adults [2] are likely due to differences in immunosuppressive therapy. Antibody induction was not performed during transplantation in most of the studies (21/34) included in the adult meta-analysis. Ten of the 34 studies reported the use of only one medication other than steroids for immunosuppression. In comparison, induction therapy was used in four of the five included studies in the present meta-analysis. Calcineurin inhibitors and either mycophenolate mofetil (MMF) or sirolimus (SRL) were included in each study. Overall, the immunosuppressive therapy was potent and therefore reduced the potential increase in the risk of AR with SAW in the recent paediatric KTx studies. It was also supported by another systematic review on very early steroid withdrawal or complete avoidance protocol for adult kidney transplant recipients, where only studies with the induction protocols was included. It demonstrated that no higher risk of acute rejection was revealed when tacrolimus (while not cyclosporine) plus MMF was used in maintenance immunosuppression [36]. The optimal SAW regimen has not been established but likely should include a calcineurin inhibitor (CNI), anti-metabolic agent or mTOR inhibitor for the maintenance of immunosuppressive therapy and likely at least a non-lymphocyte-depleting antibody for induction therapy whenever steroid-sparing strategies are to be implemented.

Importantly, the risks of NODAT and hypertension were decreased with the SAW regimen, suggesting that future cardiovascular risk would be lowered. These results are in accordance with the adult meta-analysis, which demonstrated significant reductions in the risks of hypertension, NODAT and hypercholesterolemia with the SAW protocol [2]. The overall risk of infection was unaffected by SAW in the current study, consistent with the adult meta-analysis, and the risk of cytomegalovirus (CMV) infection was also unaffected by SAW. Note that follow-up data on adverse events were recorded mainly in three studies with a total of 366 participants.

The current meta-analysis has several limitations. First, the limited number of participants in each subgroup who were stratified by factors such as the time of initiation of steroid withdrawal or developmental status did not allow for sufficient power to be achieved to identify differences between the SAW and steroid-based groups for the endpoints and could have led to substantial bias. Second, one to three years of follow-up was acceptable when evaluating the risk of AR but may have resulted in underestimation of the adverse effects of the SAW protocol (including the increased dosage of concomitant immunosuppressants or the administration of new drugs), for example, chronic rejection and CNI nephropathy. Third, the limited number of studies was not sufficient to determine publication bias.

Based on the results of this meta-analysis, we suggest that the SAW regimen is justified in select paediatric renal allograft recipients because it provides significant benefits in post-transplant growth within the first year post-withdrawal with minimal effects on the risk of AR, graft function, and graft and patient survival within 3 years post-withdrawal. These select paediatric recipients should have the following characteristics:

- Prepubertal;
- Caucasian;
- Primary disease not related to immunological factors;
- *De novo* kidney transplant recipient;
- Low PRA.

More trials are required to confirm the effect of the SAW regimen on post-transplant growth past 1 year post-withdrawal. Its effect on the growth of pubertal recipients should also be further evaluated.

## Supporting Information

S1 Fig Risk of bias summary. +/Green, low risk;? /yellow, unclear risk. Abbreviation: AR, acute rejection.
(TIF)

S2 Fig Funnel plot comparing the change in the height z-score between the SAW and SB groups of paediatric renal allograft recipients. Abbreviations: SAW, steroid avoidance or withdrawal; SB, steroid-based; SE, standard error; MD, mean difference. Produced by RevMan Version 5.2.
(TIF)

S3 Fig Funnel plot comparing the change in height z-score between the SAW and SB groups at 1 year post-withdrawal in paediatric renal allograft recipients. Abbreviations: SAW, steroid avoidance or withdrawal; SB, steroid-based; SE, standard error; MD, mean difference. Produced by RevMan Version 5.2.
(TIF)

S4 Fig Funnel plot comparing the change in height z-score between the SAW and SB groups of prepubertal paediatric renal allograft recipients. Abbreviations: SAW, steroid avoidance or withdrawal; SB, steroid-based; SE, standard error; MD, mean difference. Produced by RevMan Version 5.2.
(TIF)

S5 Fig 5 Funnel plot comparing the risk of AR between the SAW and SB groups of paediatric renal allograft recipients. Abbreviations: AR, acute rejection; SAW, steroid avoidance or withdrawal; SB, steroid-based; SE, standard error; RR, relative risk or risk ratio. Produced by RevMan Version 5.2.
(TIF)

S6 Fig Trial sequential analysis of the effect of the SAW versus SB regimen on the change in height z-score in paediatric renal allograft recipients based on five studies (n = 528). A required information size of 708 patients was calculated based on a mean difference (MD) of the ΔHSDS of 0.4 between the SAW and SB groups; a variance of 0.9; a type I error (α) of 5%; a type II error (β) of 20%; and heterogeneity of 75%. Abbreviations: ΔHSDS, change in height z-score; SAW, steroid avoidance or withdrawal; SB, steroid-based. Produced by TSA Viewer Version 0.9 Beta.
(TIF)

S7 Fig Trial sequential analysis of the effect of the SAW versus SB regimen on the change in height z-score at 1 year post-withdrawal in paediatric renal allograft recipients based on five studies (n = 528). A required information size of 601 patients was calculated based on an observed mean difference (MD) of the ΔHSDS of 0.22 between the SAW and SB groups; a variance of 0.75; a type I error (α) of 5%; a type II error (β) of 20%; and an observed heterogeneity of 19%. Abbreviations: ΔHSDS, change in height z-score; SAW, steroid avoidance or withdrawal; SB, steroid-based. Produced by TSA Viewer Version 0.9 Beta.
(TIF)

S8 Fig Trial sequential analysis of the effect of the SAW versus SB regimen on the change in height z-score in prepubertal paediatric renal allograft recipients based on five studies (n = 528). A required information size of 244 patients was calculated based on an observed mean difference (MD) of the ΔHSDS of 0.60 between the SAW and SB groups; a variance of 3.3; a type I error (α) of 5%; a type II error (β) of 20%; and an observed heterogeneity of 0%. Abbreviations: ΔHSDS, change in height z-score; SAW, steroid avoidance or withdrawal; SB, steroid-based. Produced by TSA Viewer Version 0.9 Beta.
(TIF)

S9 Fig Trial sequential analysis of the effect of the SAW versus SB regimen on acute rejection in paediatric renal allograft recipients based on five studies (n = 528). A required information size of 880 patients was calculated based on an observed acute rejection incidence of 25% in the SB group in meta-analysis (Pc); a relative risk reduction (RRR) of -40% in the SB group; a type I error (α) of 5%; a type II error (β) of 20%; and a heterogeneity of I^2^ = 25%. Abbreviations: SAW, steroid avoidance or withdrawal; SB, steroid-based. Produced by TSA Viewer Version 0.9 Beta.
(TIF)

S1 File Risk of bias in the included studies. (DOCX)

S2 File Trial sequential analysis. (DOCX)

S3 File PRISMA 2009 checklist. (DOC)

S4 File PRISMA 2009 flow diagram. (DOC)

S1 Table Search strategies. (DOCX)

S2 Table Assessment of the quality of evidence by the GRADE approach. (DOCX)
