# Supplementary figures and images for: Steroid Avoidance or Withdrawal Regimens in Paediatric Kidney Transplantation: A Meta-Analysis of Randomised Controlled Trials
Source: PLoS One. 2016 Mar 18;11(3):e0146523. doi: 10.1371/journal.pone.0146523 (PMC4798578; doi:10.1371/journal.pone.0146523)

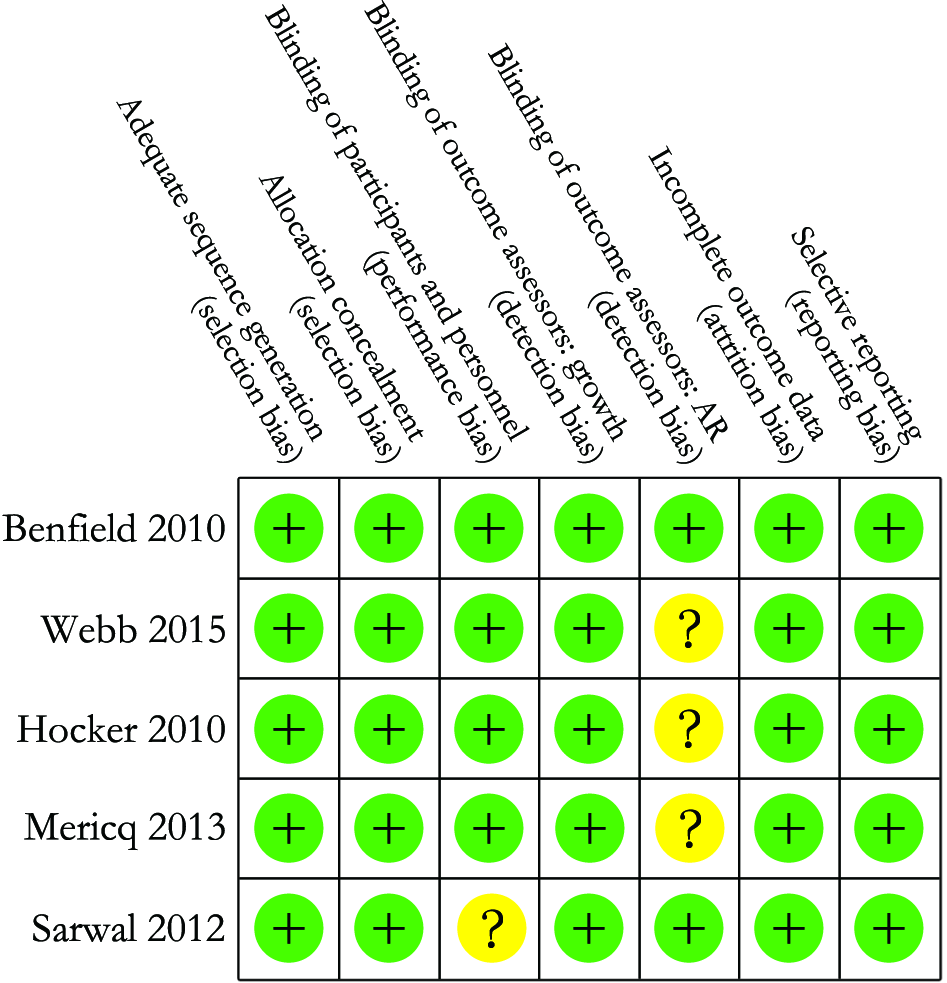

Supplement: S1 Fig — +/Green, low risk;? /yellow, unclear risk. Abbreviation: AR, acute rejection. (TIF) [file pone.0146523.s001.tif]

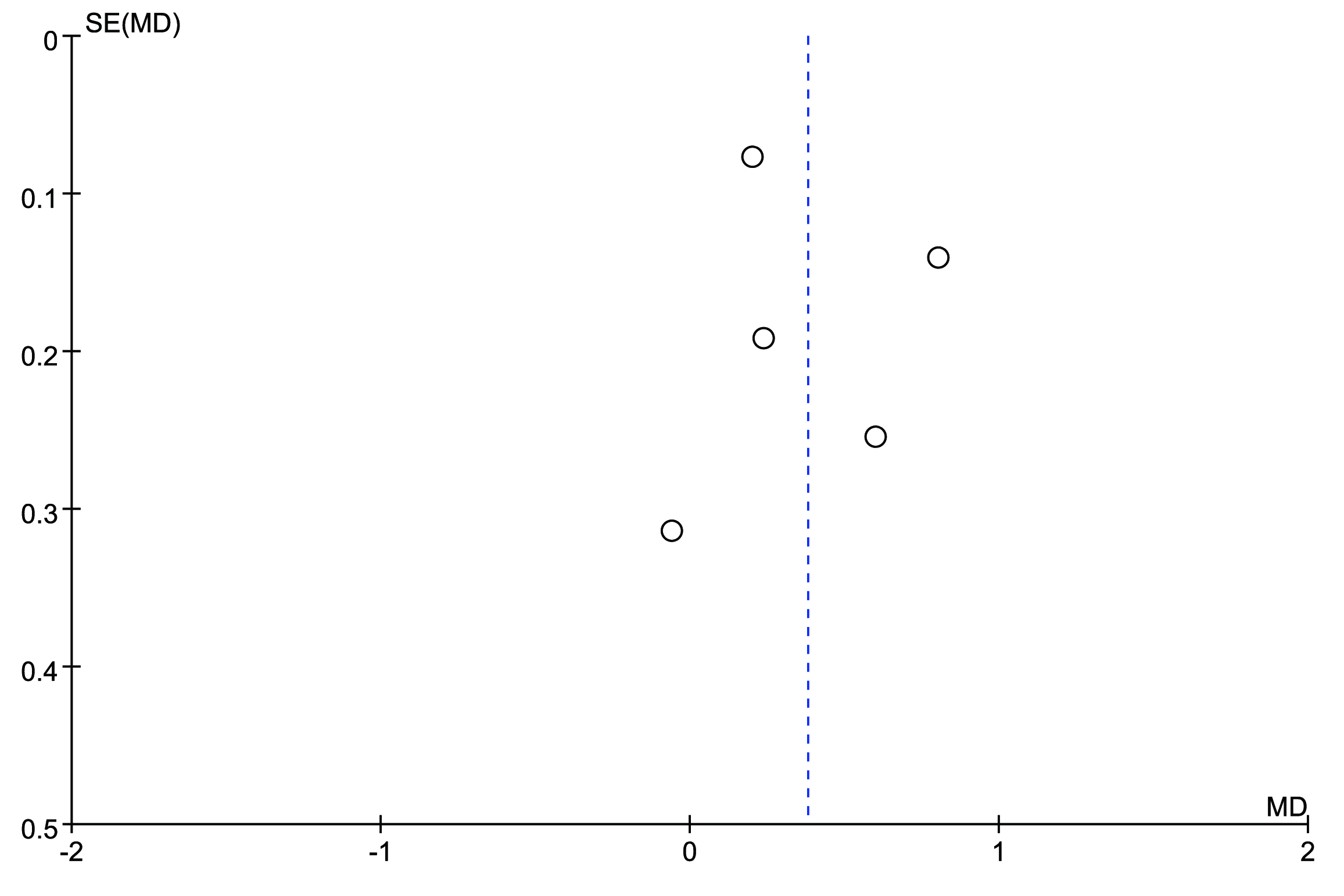

Supplement: S2 Fig — Abbreviations: SAW, steroid avoidance or withdrawal; SB, steroid-based; SE, standard error; MD, mean difference. Produced by RevMan Version 5.2. (TIF) [file pone.0146523.s002.tif]

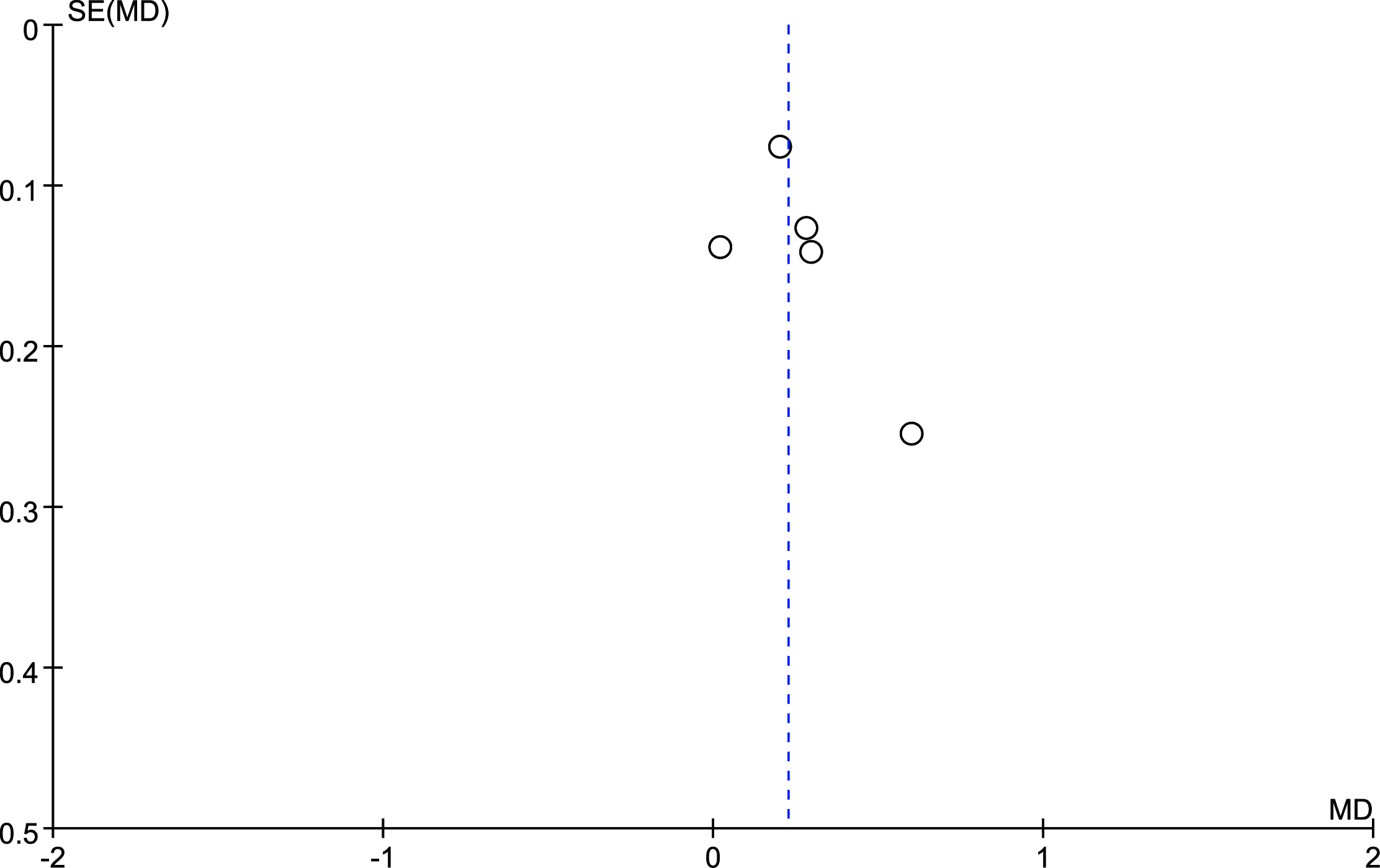

Supplement: S3 Fig — Abbreviations: SAW, steroid avoidance or withdrawal; SB, steroid-based; SE, standard error; MD, mean difference. Produced by RevMan Version 5.2. (TIF) [file pone.0146523.s003.tif]

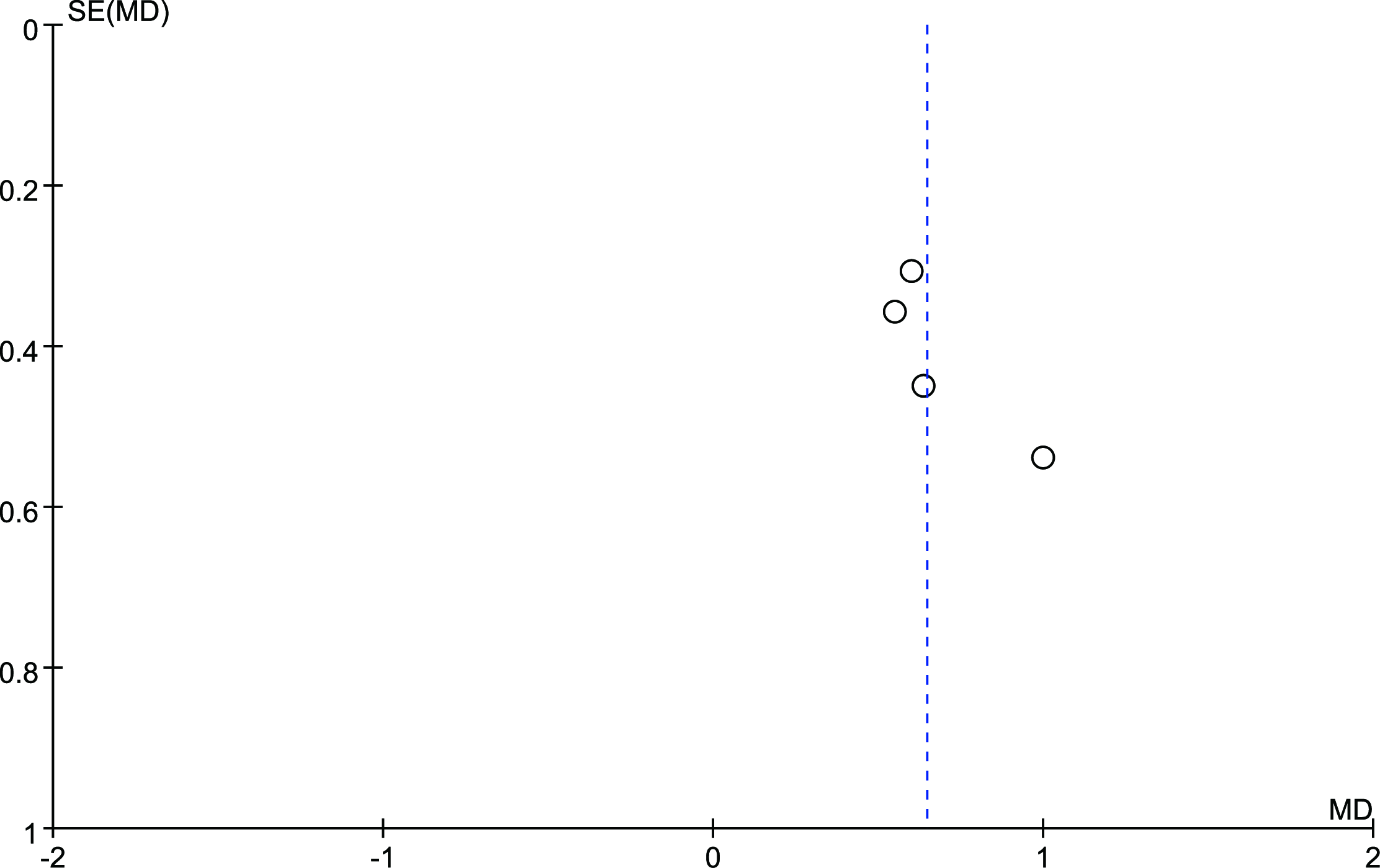

Supplement: S4 Fig — Abbreviations: SAW, steroid avoidance or withdrawal; SB, steroid-based; SE, standard error; MD, mean difference. Produced by RevMan Version 5.2. (TIF) [file pone.0146523.s004.tif]

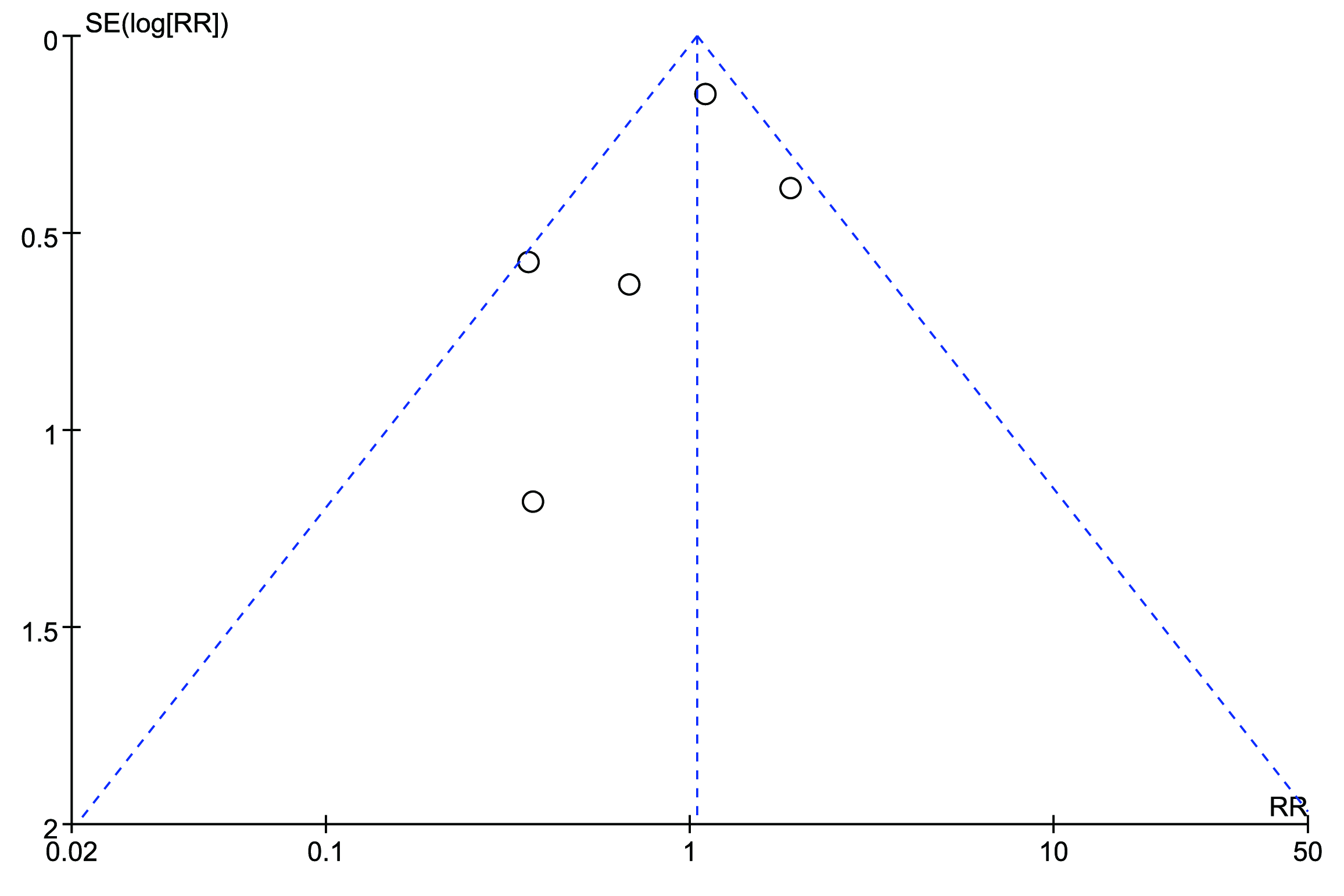

Supplement: S5 Fig — Abbreviations: AR, acute rejection; SAW, steroid avoidance or withdrawal; SB, steroid-based; SE, standard error; RR, relative risk or risk ratio. Produced by RevMan Version 5.2. (TIF) [file pone.0146523.s005.tif]

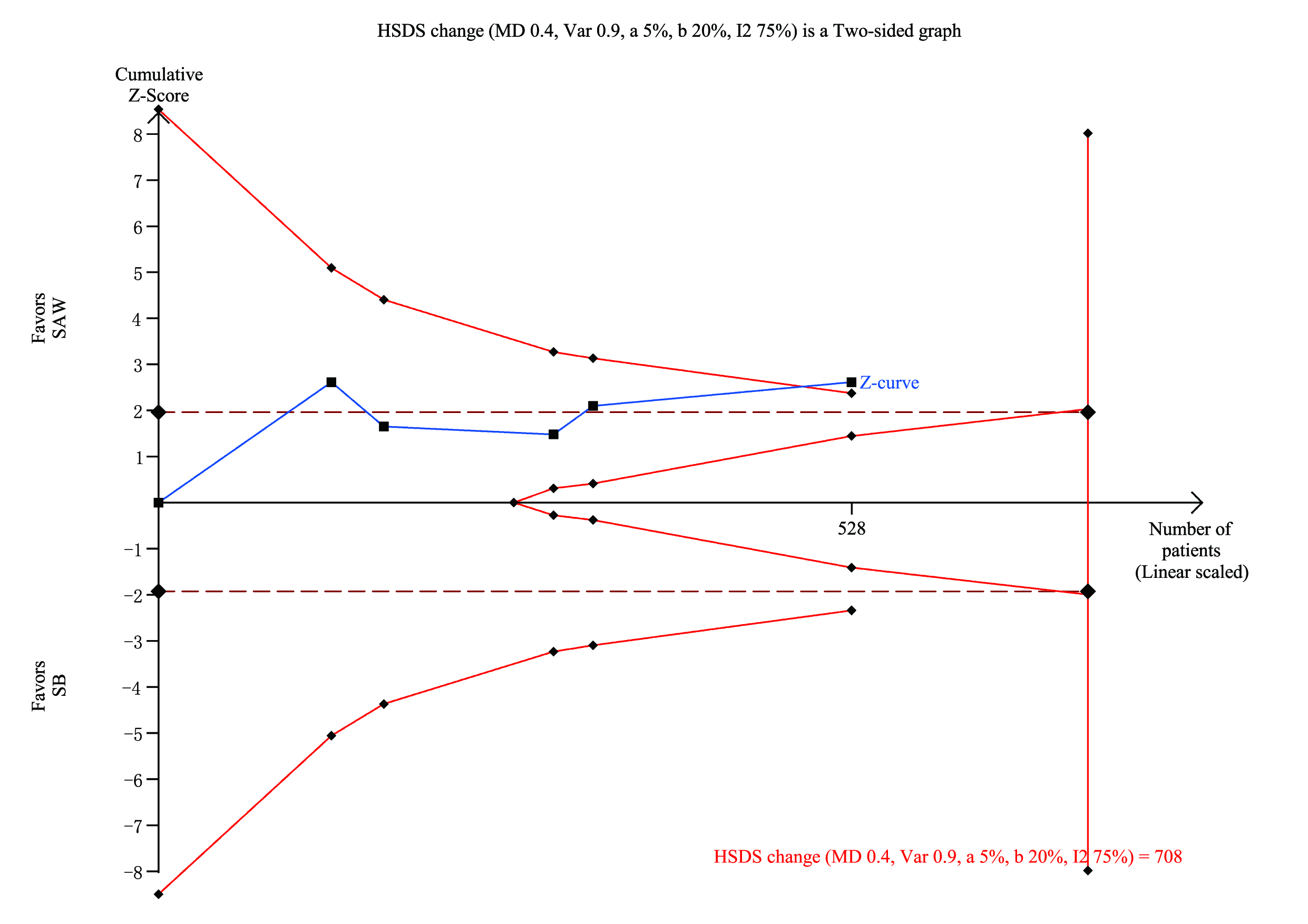

Supplement: S6 Fig — A required information size of 708 patients was calculated based on a mean difference (MD) of the ΔHSDS of 0.4 between the SAW and SB groups; a variance of 0.9; a type I error (α) of 5%; a type II error (β) of 20%; and heterogeneity of 75%. Abbreviations: ΔHSDS, change in height z-score; SAW, steroid avoidance or withdrawal; SB, steroid-based. Produced by TSA Viewer Version 0.9 Beta. (TIF) [file pone.0146523.s006.tif]

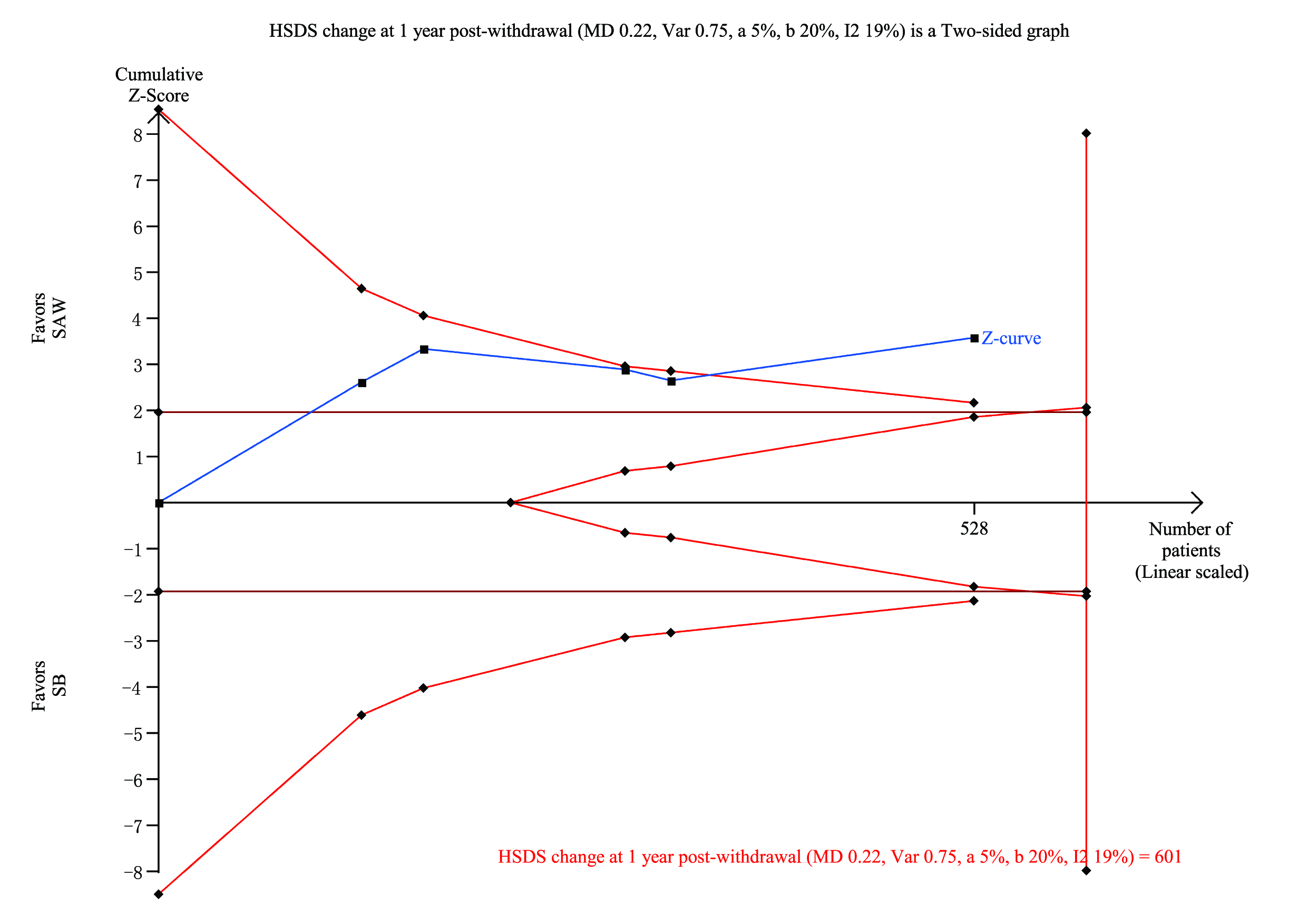

Supplement: S7 Fig — A required information size of 601 patients was calculated based on an observed mean difference (MD) of the ΔHSDS of 0.22 between the SAW and SB groups; a variance of 0.75; a type I error (α) of 5%; a type II error (β) of 20%; and an observed heterogeneity of 19%. Abbreviations: ΔHSDS, change in height z-score; SAW, steroid avoidance or withdrawal; SB, steroid-based. Produced by TSA Viewer Version 0.9 Beta. (TIF) [file pone.0146523.s007.tif]

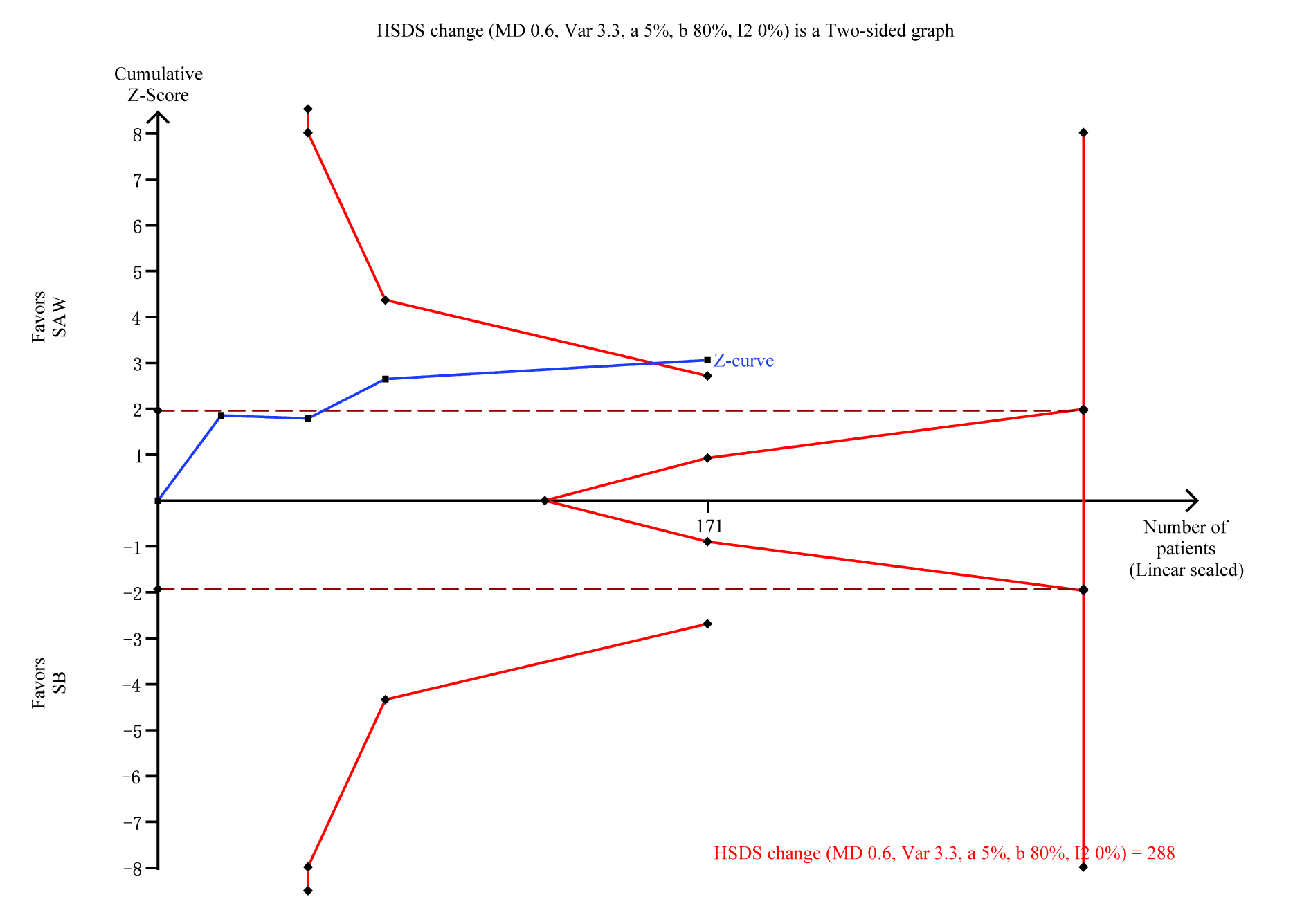

Supplement: S8 Fig — A required information size of 244 patients was calculated based on an observed mean difference (MD) of the ΔHSDS of 0.60 between the SAW and SB groups; a variance of 3.3; a type I error (α) of 5%; a type II error (β) of 20%; and an observed heterogeneity of 0%. Abbreviations: ΔHSDS, change in height z-score; SAW, steroid avoidance or withdrawal; SB, steroid-based. Produced by TSA Viewer Version 0.9 Beta. (TIF) [file pone.0146523.s008.tif]

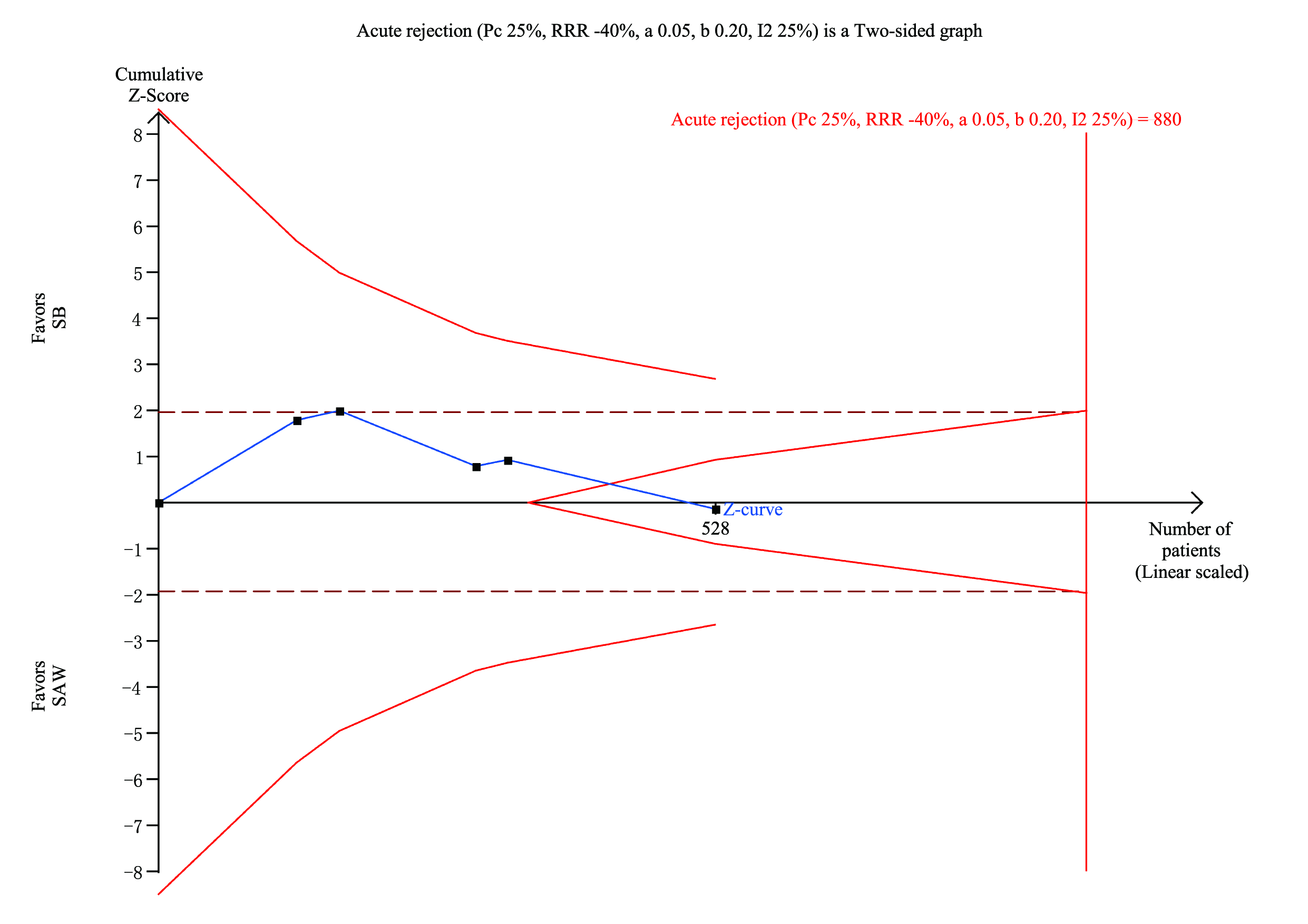

Supplement: S9 Fig — A required information size of 880 patients was calculated based on an observed acute rejection incidence of 25% in the SB group in meta-analysis (Pc); a relative risk reduction (RRR) of -40% in the SB group; a type I error (α) of 5%; a type II error (β) of 20%; and a heterogeneity of I2 = 25%. Abbreviations: SAW, steroid avoidance or withdrawal; SB, steroid-based. Produced by TSA Viewer Version 0.9 Beta. (TIF) [file pone.0146523.s009.tif]
