# Supplementary material for: Steroid Avoidance or Withdrawal Regimens in Paediatric Kidney Transplantation: A Meta-Analysis of Randomised Controlled Trials
Source: PLoS One. 2016 Mar 18;11(3):e0146523. doi: 10.1371/journal.pone.0146523 (PMC4798578; doi:10.1371/journal.pone.0146523)
Supplement: S1 File — (DOCX) [file pone.0146523.s010.docx]

Risk of bias in the included studies

The summary of risk of bias is presented in S1 Fig.

Allocation

All five studies reported the use of a randomised design. Further investigation revealed that only three studies employed random sequence generation implemented by software or a program [1-5], whereas the other two studies simply described the process as randomised [6-8], which we considered scarcely sufficient to avoid a high risk of bias. Overall, all five studies were at a low risk of bias due to inadequate sequence generation. Four of the five studies reported an adequate method of allocation concealment using sealed sequentially numbered randomisation containers or a web-based system [1-6, 8]. In the other study, assignment was concealed by the method of central randomisation [7], thereby meeting the minimal criteria for adequate allocation concealment. In summary, the risk of selection bias was low across studies.

Blinding

M. R. Benfield et al. blinded participants and personnel in a placebo-controlled fashion [7]. The other four studies were all open-labelled [1-6,8]. Growth hormone therapy was prohibited during three of the four open-labelled studies [1,2,4,6,8]. The other study did not report on the use of growth hormone as a concomitant medication [3,5], which was judged as an unclear risk of performance bias and which likely affected linear growth due to the probable unbalanced use of growth hormone. AR was unlikely to be affected by the lack of blinding of participants and personnel and was thus at a low risk of performance bias.

Information was not provided about whether the investigators who were engaged in the measurement of height were masked during all clinical trials [1-8]. Linear growth is an objective outcome that is unlikely to be influenced by a lack of blinding of the outcome assessor; thus, the risk of detection bias was low. The pathologists who were responsible for the judgement of biopsy-proven acute rejection (AR) were blinded in two studies [3,5,7], whereas information was not provided in the other three studies [1,2,4,6,8]; thus, the risk of detection bias was unclear.

Incomplete outcome data

V. Mericq et al. and M. R. Benfield et al. reported no missing outcome data after randomisation [2,7]. N. J. Webb et al. [1,4] reported balanced numbers of missing data between the steroid avoidance or withdrawal (SAW) and steroid-based (SB) groups, with similar reasons for missing data between the groups. In the other two studies with attrition of participants, intention-to-treat (ITT) analysis was performed [3,5,6,8]. In summary, all five studies were at a low risk of attrition bias.

Selective reporting

The protocols of three studies were accessible in a clinical trials registry [2,3,5,6,8], and those of the other two were not accessible [1,4,7]; however, it was clear that the published reports included all expected outcomes. All enrolled studies were at a low risk of reporting bias.

Summary assessment

The mean difference (MD) in the change in the height Z-score change (ΔHSDS) was at a low risk of bias in all five studies; thus, the summary effect was also at a low risk of bias. The relative risk of AR was at a low risk of bias in two studies and at an unclear risk in three studies, suggesting plausible bias in the summary effect.

References

1. Webb NJA, Douglas SE, Rajai A, et al. Corticosteroid-free kidney transplantation improves growth: 2-year follow-up of the TWIST Randomized Controlled Trial. Transplantation 2015; 99**:** 1178-1185

2. Mericq V, Salas P, Pinto V, et al. Steroid withdrawal in pediatric kidney transplant allows better growth, lipids and body composition: a randomized controlled trial. Horm Res Paediatr 2013; 79**:** 88-96

3. Sarwal MM, Ettenger RB, Dharnidharka V, et al. Complete steroid avoidance is effective and safe in children with renal transplants: a multicenter randomized trial with three-year follow-up. Am J Transplant 2012; 12**:** 2719-2729

4. Grenda R, Watson A, Trompeter R, et al. A randomized trial to assess the impact of early steroid withdrawal on growth in pediatric renal transplantation: the TWIST study. Am J Transplant 2010; 10**:** 828-836

5. Sarwal M, Benfield M, Ettenger R, et al. One year results of a prospective, randomized, multicenter trial of steroid avoidance in pediatric renal transplantation. Am J Transplant 2008; 82**:** 192-193

6. Hocker B, Weber LT, Feneberg R, et al. Improved growth and cardiovascular risk after late steroid withdrawal: 2-year results of a prospective, randomised trial in paediatric renal transplantation. Nephrol Dial Transplant 2010; 25**:** 617-624

7. Benfield MR, Bartosh S, Ikle D, et al. A randomized double-blind, placebo controlled trial of steroid withdrawal after pediatric renal transplantation. Am J Transplant 2010; 10**:** 81-88

8. Höcker B, Weber LT, Feneberg R, et al. Prospective, randomized trial on late steroid withdrawal in pediatric renal transplant recipients under cyclosporine microemulsion and mycophenolate mofetil. Transplantation 2009; 87: 934-941
