# Supplementary material for: Steroid Avoidance or Withdrawal Regimens in Paediatric Kidney Transplantation: A Meta-Analysis of Randomised Controlled Trials
Source: PLoS One. 2016 Mar 18;11(3):e0146523. doi: 10.1371/journal.pone.0146523 (PMC4798578; doi:10.1371/journal.pone.0146523)
Supplement: S2 File — (DOCX) [file pone.0146523.s011.docx]

Trial sequential analysis

Subjects and Methods

Trial sequential analysis (TSA) was performed to control the risk of errors for a given outcome and to help clarify whether additional trials are required [1-4]. We calculated the diversity-adjusted required information size (IS) and applied trial sequential monitoring boundaries with TSA Viewer Version 0.9 Beta ((c) Copyright Copenhagen Trial Unit, 2011). The IS was based on an alpha of 0.05 and a beta of 0.20 (power of 80%). The difference in and variance of the intervention effect between studies in terms of continuous data and the incidence in the control arm and relative risk reduction in terms of dichotomous data were determined by the outcomes for the included studies that were at a low risk of bias, as well as from empirical evidence. The heterogeneity between studies, expressed as I^2^, was presumed to be the observed value of 25%, 50% or 75% in sensitivity analysis when calculating the IS. TSA was performed for all outcome measures.

Results

The required IS for the ΔHSDS was 708 patients (S6 Fig). Although this IS was not reached, the cumulative Z-curve reached the efficacy area, indicating that there was sufficient evidence to make a conclusion. The IS for the ΔHSDS at 1 year post-withdrawal was 601 patients (S7 Fig 7), and although it was not reached, the cumulative Z-curve reached the efficacy area. The IS for the ΔHSDS at over 1 year post-withdrawal was 2166 patients (MD=0.38, variance (Var)=2.09, I^2^=79%), and neither the IS nor the futility area was reached. The IS for the ΔHSDS in the prepubertal recipients was 288 patients (S8 Fig), and although it was not reached, the cumulative Z-curve reached the efficacy area. The IS for the ΔHSDS in the pubertal recipients was 1046 patients (MD=0.3, Var=2.64, I^2^=12%), and neither the IS nor the futility area was reached.

The IS for AR was 880 patients, and although it was not reached, the cumulative Z-curve reached the futility area, indicating that we could reject a 40% increase in the relative risk (S9 Fig). TSA indicated that the evidence was sufficient for AR at 1 year post-withdrawal but insufficient for AR past 1 year post-withdrawal.

References

1. Kristian Thorlund JEJW, Georgina Imberger CG. User manual for Trial Sequential Analysis (TSA).: Copenhagen Trial Unit; 2011. Available from <http://www.ctu.dk/tsa/files/tsa_manual.pdf>

2. Castellini G, Gianola S, Banzi R, et al. Constraint-induced movement therapy: trial sequential analysis applied to Cochrane collaboration systematic review results. Trials 2014; 15: 512

3. Penninga L, Penninga EI, Moller CH, Iversen M, Steinbruchel DA, Gluud C. Tacrolimus versus cyclosporin as primary immunosuppression for lung transplant recipients. Cochrane Database Syst Rev 2013; 5: D8817

4. Wetterslev J, Thorlund K, Brok J, Gluud C. Trial sequential analysis may establish when firm evidence is reached in cumulative meta-analysis. J Clin Epidemiol 2008; 61: 64-75
