# Supplementary material for: Steroid Avoidance or Withdrawal Regimens in Paediatric Kidney Transplantation: A Meta-Analysis of Randomised Controlled Trials
Source: PLoS One. 2016 Mar 18;11(3):e0146523. doi: 10.1371/journal.pone.0146523 (PMC4798578; doi:10.1371/journal.pone.0146523)
Supplement: S4 File — (DOC) [file pone.0146523.s013.doc]

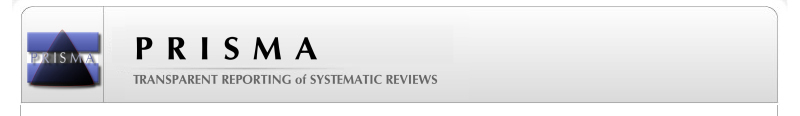
**PRISMA 2009 Flow Diagram**

**Screening**

**Included**

**Eligibility**

**Identification**

Records identified through database searching (Pubmed, Embase, Cochrane library)
(n = 3845 )

Additional records identified through other sources (BIOSIS previews, clinicaltrials.gov)
(n = 496 )

Records after duplicates removed
(n = 548 )

Records screened
(n = 3793 )

Records excluded based on titles and abstracts
(n = 3608 )

Full-text articles assessed for eligibility
(n = 185 )

Full-text articles excluded, with reasons
(n = 175 )

- Ineligible populations (n=85)
- Not RCT (n=64)
- Not related to SAW (n=7)
- Same study (n=19)
- Ongoing RCT (n=1)
- Abstract only (n=1)

Studies included in qualitative synthesis
(8 articles from 5 RCTs)

Studies included in quantitative synthesis (meta-analysis)
(8 articles from 5 RCTs)
