# Supplementary material for: Steroid Avoidance or Withdrawal Regimens in Paediatric Kidney Transplantation: A Meta-Analysis of Randomised Controlled Trials
Source: PLoS One. 2016 Mar 18;11(3):e0146523. doi: 10.1371/journal.pone.0146523 (PMC4798578; doi:10.1371/journal.pone.0146523)
Supplement: S1 Table — (DOCX) [file pone.0146523.s014.docx]

**S1 Table Search strategies**

| **Database** | **Search terms** |
| --- | --- |
| PubMed | 1. ((glucocorticoids[mesh] or steroids[mesh] or prednisone[mesh] or "adrenal cortex hormones"[mesh] or prednisolone[mesh] or methylprednisolone[mesh]) or glucocorticoid* or glucorticoid* or glucocorticosteroid* or corticosteroid* or corticoid* or steroid* or "adrenal cortex hormone" or "adrenal cortex hormones" or cortisone or prednisone or prednisolone or methylprednisolone or medrol)  2. ((pediatrics[mesh] or child[mesh] or infant[mesh] or adolescent[mesh]) or (neonat* or newborn* or infan* or toddler* or preschool* or school* or child* or pediatr* or paediatr* or kid or kids or baby or babies or adolescen* or teen* or youth or youths or minors))  3. ("kidney transplantation"[mesh] or (kidney transplant*) or (kidney graft*) or (kidney allograft*) or (renal transplant*) or (renal graft*) or (renal allograft*))  4. ((randomized controlled trial [pt] or controlled clinical trial [pt] or randomized [tiab] or placebo [tiab] or drug therapy [sh] or randomly [tiab] or trial [tiab] or groups [tiab]) not (animals [mh] not humans [mh]))  5. #1 and #2 and #3 and #4 |
| Embase | 1. (glucocorticoid/exp or steroid/exp or prednisone/exp or corticosteroid/exp or prednisolone/exp or methylprednisolone/exp or (glucocorticoid* or glucorticoid* or glucocorticosteroid* or corticosteroid* or corticoid* or steroid* or "adrenal cortex hormone" or "adrenal cortex hormones" or cortisone or prednisone or prednisolone or methylprednisolone or medrol))  2. (pediatrics/exp or child/exp or infant/exp or adolescence/exp or puberty/exp or (neonat* or newborn* or infan* or toddler* or preschool* or school* or child* or pediatr* or paediatr* or kid or kids or baby or babies or adolescen* or teen* or youth or youths or minors or pubert*))  3. (“kidney transplantation”/exp or (kidney transplant*) or (kidney graft*) or (kidney allograft*) or (renal transplant*) or (renal graft*) or (renal allograft*))  4. ((“crossover-procedure”/exp or “double-blind procedure”/exp or “randomized controlled trial”/exp or “single-blind procedure”/exp) or (random$ or factorial$ or crossover$ or (cross over$) or cross-over$ or placebo$ or (doubl$ near/5 blind$) or (singl$ near/5 blind$) or assign$ or allocate$ or volunteer$))  5. #1 and #2 and #3 and #4 |
| Cochrane Library | 1. (([mh glucocorticoids] or [mh steroids] or [mh prednisone] or [mh "adrenal cortex hormones"] or [mh prednisolone] or [mh methylprednisolone]) or glucocorticoid***** or glucorticoid***** or glucocorticosteroid***** or corticosteroid***** or corticoid***** or steroid***** or "adrenal cortex hormone" or "adrenal cortex hormones" or cortisone or prednisone or prednisolone or methylprednisolone or medrol)  2. (([mh pediatrics] or [mh child] or [mh infant] or [mh adolescent]) or (neonat* or newborn* or infan* or toddler* or preschool* or school* or child* or pediatr* or paediatr* or kid or kids or baby or babies or adolescen* or teen* or youth or youths or minors))  3. ([mh "kidney transplantation"] or (kidney transplant*) or (kidney graft*) or (renal transplant*) or (renal graft*))  4. #1 and #2 and #3 |
| BIOSIS Previews | 1. ts=(glucocorticoid* or glucorticoid* or glucocorticosteroid* or corticosteroid* or corticoid* or steroid$ or adrenal cortex hormone* or cortisone or prednisone or prednisolone or methylprednisolone or medrol)  2. ts=(neonat* or newborn* or infan* or toddler* or preschool* or school* or child* or pediatr* or paediatr* or kid or kids or baby or babies or adolescen* or teen* or youth or youths or minors or pubert*)  3. ts=((kidney transplant*) or (kidney graft*) or (kidney allograft*) or (renal transplant*) or (renal graft*) or (renal allograft*))  4. #1 and #2 and #3 (Refined by: document types: ( meeting or letter or meeting paper ) ) |
| Clinicaltrials.gov | 1. (glucocorticoid* or glucorticoid* or glucocorticosteroid* or corticosteroid* or corticoid* or steroid* or "adrenal cortex hormone" or "adrenal cortex hormones" or cortisone or prednisone or prednisolone or methylprednisolone or medrol) [all-fields]  2. ((kidney transplant*) or (kidney graft*) or (kidney allograft*) or (renal transplant*) or (renal graft*) or (renal allograft*)) [all-fields]  3. "child" [age-group]  4. #1 and #2 and #3 |
