# Supplementary material for: Steroid Avoidance or Withdrawal Regimens in Paediatric Kidney Transplantation: A Meta-Analysis of Randomised Controlled Trials
Source: PLoS One. 2016 Mar 18;11(3):e0146523. doi: 10.1371/journal.pone.0146523 (PMC4798578; doi:10.1371/journal.pone.0146523)
Supplement: S2 Table — (DOCX) [file pone.0146523.s015.docx]

**S2 Table Assessment of the quality of evidence by the GRADE approach**

| **Outcome** | **Limitations in design and implementation** | **Indirectness of evidence** | **Unexplained heterogeneity** | **Imprecision** | **High probability of publication bias** | **Quality of evidence (GRADE)** |
| --- | --- | --- | --- | --- | --- | --- |
| ΔHSDS | No | No | No | No | No | High |
| ΔHSDS at 1 year post-withdrawal | No | No | No | No | No | High |
| ΔHSDS over 1 year post-withdrawal | No | No | Yes (-1) | No | No | Moderate |
| ΔHSDS in prepubertal | No | No | No | No | No | High |
| ΔHSDS in prepubertal at 1 year post-withdrawal | No | No | No | No | No | High |
| ΔHSDS in pubertal | No | No | No | No | No | High |
| Acute rejection | No | No | No | No | No | High |
| AR at 1 year post-withdrawal | No | No | No | No | No | High |
| AR over 1 year post-withdrawal | No | No | No | No | No | High |
| eGFR at 6 months post-withdrawal | No | No | No | No | No | High |
| eGFR at 1 year post-withdrawal | No | No | No | No | No | High |
| eGFR at 2 years post-withdrawal | No | No | No | No | No | High |
| NODAT | No | No | No | No | No | High |
| Hypertension | No | No | No | No | No | High |
| Infection | No | No | No | No | No | High |
| CMV infection | No | No | No | Yes (-1) | No | Moderate |
| PTLDs | No | No | No | Yes (-1) | No | Moderate |
| DGF | No | No | Yes (-1) | Yes (-1) | No | Low |
| Anaemia | No | No | Yes (-1) | Yes (-1) | No | Low |
